# An automated quantitative image analysis pipeline of *in vivo* oxidative stress and macrophage kinetics

**DOI:** 10.14440/jbm.2018.259

**Published:** 2018-11-07

**Authors:** Andre D. Paredes, David Benavidez, Jun Cheng, Steve Mangos, Michael Donoghue, Amelia Bartholomew

**Affiliations:** 1 Richard and Loan Hill Department of Bioengineering, University of Illinois, Chicago, IL 60612, USA; 2 Department of Surgery, University of Illinois, Chicago, IL 60612, USA; 3 Department of Internal Medicine, Rush University, Chicago, IL 60612, USA; 4 Donoghue Chiropractic, Lincolnshire, IL 60069, USA

**Keywords:** cell kinetics, oxidative stress, quantitative image analysis, time-lapse imaging, zebrafish

## Abstract

Macrophage behavior is of great interest in response to tissue injury and promotion of regeneration. With increasing numbers of zebrafish reporter-based assays, new capabilities now exist to characterize macrophage migration, and their responses to biochemical cues, such as reactive oxygen species. Real time detection of macrophage behavior in response to oxidative stress using quantitative measures is currently beyond the scope of commercially available software solutions, presenting a gap in understanding macrophage behavior. To address this gap, we developed an image analysis pipeline solution to provide real time quantitative measures of cellular kinetics and reactive oxygen species content *in vivo* after tissue injury. This approach, termed Zirmi, differs from current software solutions that may only provide qualitative, single image analysis, or cell tracking solutions. Zirmi is equipped with user-defined algorithm parameters to customize quantitative data measures with visualization checks for an analysis pipeline of time-based changes. Moreover, this pipeline leverages open-source PhagoSight, as an automated keyhole cell tracking solution, to avoid parallel developments and build upon readily available tools. This approach demonstrated standardized space- and time-based quantitative measures of (1) fluorescent probe based oxidative stress and (2) macrophage recruitment kinetic based changes after tissue injury. Zirmi image analysis pipeline performed at execution speeds up to 10-times faster than manual image-based approaches. Automated segmentation methods were comparable to manual methods with a DICE Similarity coefficient > 0.70. Zirmi provides an open-source, quantitative, and non-generic image analysis pipeline. This strategy complements current wide-spread zebrafish strategies, for automated standardizations of analysis and data measures.

## INTRODUCTION

The speed of inflammation is highly influenced by the magnitude of oxidative stress and the number and function of wound macrophages [1,2]. Macrophages have a diverse response to oxidative stress in health and disease that underscores a need to better understand how tissue level changes of oxidative stress can enhance or impair injury responses. Zebrafish can be bred to express fluorescent transgenic inflammatory cells such as macrophages and can be imaged in a whole organism approach in real time due to their translucent phenotypical characteristics [3]. The conservation between zebrafish and human pathways has been verified extensively [4] and has led to high impact discoveries in post-injury leukocyte recruitment and oxidative stress [5]. Therefore, zebrafish image-based analysis is a targeted strategy to characterize biological phenomena for more comprehensive insights into human inflammatory responses. Currently, independent gains in methodologies have been made to fluorescently evaluate oxidative stress [6] and macrophage migration [7] in zebrafish after injury. However, no parallel analysis pipeline exists to leverage these *in vivo* image based evaluations simultaneously over time.

Current technologies that quantify oxidative stress responses are often accomplished using qualitative assessments that may not be measured in real time. Reactive oxygen species (ROS), commonly accepted as measures for evaluating oxidative stress, are typically characterized by qualitative measures or *ex vivo* static endpoints [6,8]. *In vivo*, tailored, quantitative analyses are not easily accomplished by commercial solutions; there is an established need for image-based pipeline analysis [9-12]. In contrast, macrophage tracking image processing algorithms and commercial software packages are more advanced. Imaging tools and software such as ImageJ^®^, Fiji^®^, Volocity^®^, Metamorph^®^ and Photoshop^®^ have been used to map morphological behavior following zebrafish fin injury [7,13-16]. Zebrafish cell tracking software strategies have yielded discrete analyses on relatively few fish (*n* < 3), short time segments, large sampling frequencies (3–10 min), or relatively small populations of cells (~10 cells per fish). Higher throughput analyses tracking larger cell numbers and analyzing longer imaging time segments have been technologically prohibitive; substantive time-consuming effort has been required to comprehensively and accurately format time-based changes during manual modes of tracking and data management. Many image processing strategies do not provide open-source transferable analysis solutions; non-standard data formats, for example, can prohibit broad implementation [17]. A recent survey of the scientific community querying automated processing strategies for zebrafish image data led to a call for image analysis software to be transparent, user-friendly, and readily available [9]. To address the need for parallel and quantitative time-based changes of both ROS and cell tracking measures, a standardized, automated analysis approach would be required. The motivation for this work was to provide a quantitative, high throughput, automated analysis pipeline, capable of simultaneously measuring both oxidative stress and macrophage kinetics in real time.

Zirmi (Zebrafish inflammatory reactive oxygen species- and macrophage kinetics-based image analysis) software was developed on the MATLAB platform to provide (1) automated image processing and (2) quantitative strategies (3) to manage and export data in reproducible formats, (4) leveraging existing image processing open-source software tools. One main advantage for using the Zirmi analysis pipeline is that it avoids parallel developments by building upon established, automated, and readily available PhagoSight pre-processing and keyhole tracking algorithms [18]. Another advantage of Zirmi analysis is that it introduces a user-customizable mode to select regions and cell tracks for standardized formats of quantitative measures. Lastly, Zirmi provides automated data management which is available opensource and can be readily amended to improve throughput and accessibility issues. We tested this analysis pipeline, according to wide-spread zebrafish preparation and imaging strategies, to acquire standardized quantitative data measures of oxidative stress and cell kinetics based on the severity of wounding. Our study demonstrates that the Zirmi analysis pipeline solution facilitates processing gains in time, reproducibility, and data measures that support both oxidative stress and cell kinetic quantitative evaluations.

## METHODS

### Zebrafish assay preparation

The Tg(mpeg:dendra2) zebrafish line, in which the promoter mpeg was used to drive dendra2 expression in macrophages [19], was purchased through ZIRC (Cat. # ZL10389). Housing, water quality, spawning, and maintenance were performed according to standard protocols [6]. All procedures and animal conditions described herein were approved by the Animal Care Committee at the University of Illinois at Chicago.

**Figure 1.**
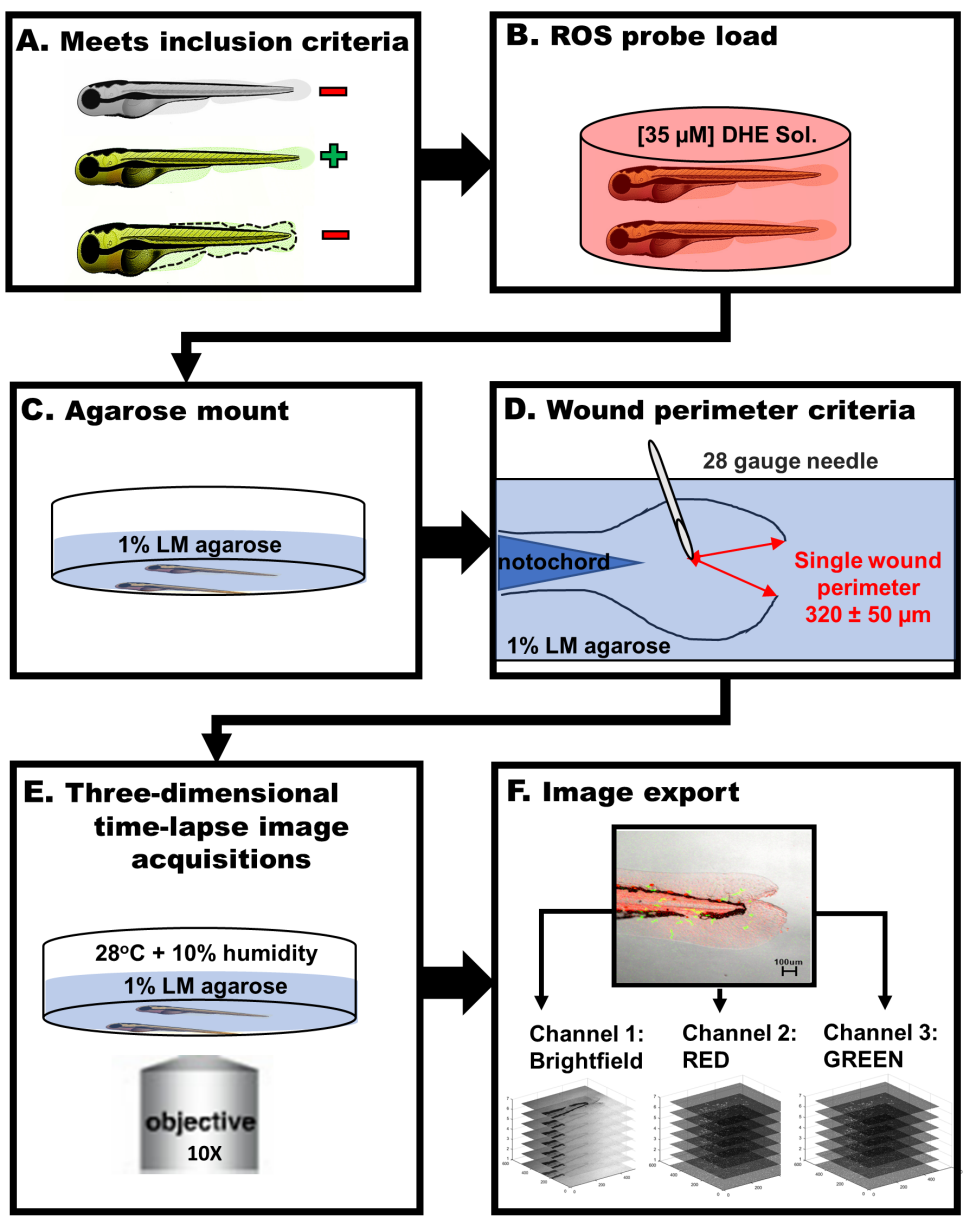
Zebrafish image-based assay workflow schematic. **A.** Zebrafish larvae selected for image-based assay had to meet an inclusion criterion based on physiological and fluorescence quality. **B.** Fish selected were loaded with reactive oxygen species probe solution, Dihydroethidium [35 μM], which was optimized according to microscope setup. **C.** Fish were then mounted laterally in 1% low melting temperature agarose as flat as possible. **D.** Once mounted, fish were subjected to specific injury resulting in “V” shaped wound perimeter (red) with a sterile 28-gauge needle. **E.** Minutes post injury fish were placed in a controlled 28°C temperature and 10% humidity environment and imaged in a 3D+t manner at 10x magnification to capture cell migration. **F.** Greyscale 3D+t digital images were exported into directories by channel: Brightfield, RED, and GREEN.

Fish selection: Naturally dechorionated larvae at 3 dpf (days post-fertilization) were placed in 75 mm glass dishes with 5 ml of E3 media. Animals that did not meet uniformity in wound size, were absent of fluorescence, or demonstrated small breaks or nicks in the embryonic outline were euthanized and not included in the experiments (**Fig. 1A**).

ROS probe loading: Dihydroethidium (DHE, Sigma) dye was reconstituted in 1 ml of anhydrous dimethyl sulfoxide (DMSO, Sigma-Aldrich) at 10 mM and stored in aliquots. Zebrafish larvae (3 dpf) were transferred, in groups of 8–15, to a 35 mm diameter glass bottom dish (MATEK), loaded [6] with 35 μM DHE solution in 1× Dulbecco’s Phosphate Buffer Saline, incubated for 40 min, transferred to a separate 75 mm glass dish, and washed with 28°C pre-warmed E3 media to remove excess DHE (**Fig. 1B**).

Mounting and wound placement: After ROS loading, larvae zebrafish were anesthetized (168 mg/L Tricaine, Sigma), mounted laterally, and immobilized with 1% low melting point agarose solution (**Fig. 1C**); zebrafish underwent single or dual caudal fin incisions, initiated ~50–100 μm from notochord and extend to the end of the caudal fin *via* 28-gauge sterile needle (**Fig. 1D**).

### Imaging

A Zeiss LSM 510 confocal microscope was used with an NA 0.75/10× objective to image the zebrafish in time-lapse conditions. Two fluorescent channels (ch) were filtered, *via* ZEN^®^ software, with the following excitations: 488 nm at 0.80 mW and 561 nm at 0.08 mW for dendra2 and ethidium, respectively. Three-dimensional time-lapse (3D+t) images were taken in 60 s intervals from 25 to 130 min post injury (MPI). The lateral pixel resolution was 1.64 μm and the image dimensions were 512 × 512 pixels. Using microscope programmed automation, up to 8 fish were imaged *per se*ssion with at least one non-wound for group baseline comparative analysis. Z-stacks were comprised of 7 frames with 10 μm spacing along the Z axis. The typical larval zebrafish (3 dpf) caudal fin demonstrated a depth approaching 100 μm in thickness, while macrophages demonstrated a depth ranging from 10 μm to 20 μm. Fluorescent macrophages were detected with various optical sections, overcoming the limitations of the NA 0.75/10× objective depth of focus of ~10 μm. Zebrafish Larvae were maintained in an enclosed microscope stage which maintained a uniform condition of 28°C and 10% Humidity (**Fig. 1E**). Greyscale images with 16 bits per pixel (BPP) were acquired and exported in TIFF file format from ZEN^®^ software (**Fig. 1F**). The TIFF files were stored within a unique experiment labeled directory. Raw images were exported to folders labeled by channel name: grayscale z-stack bright field (channel 1: BF); Texas Red fluorescent z-stack 561 nm (channel 2: RED); GFP fluorescent z-stack 488 nm (channel 3: GREEN).

### Overview of software

**Figure 2.**
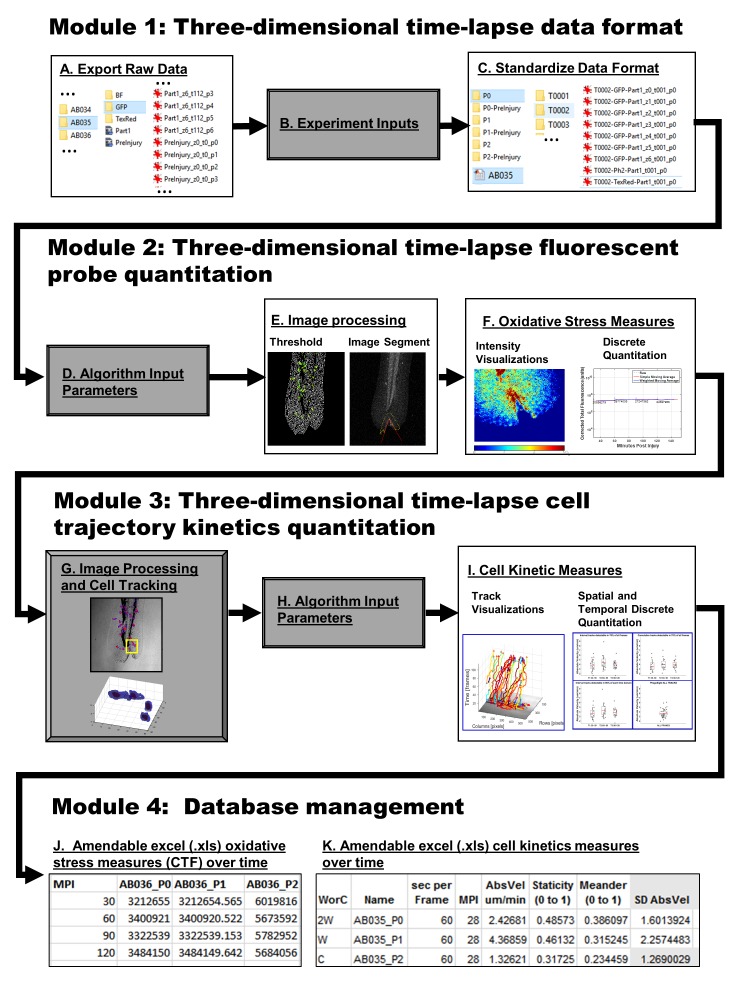
Zirmi software image analysis pipeline schematic. Zirmi software progresses raw data through 4 modules in an automated fashion. In module 1, three-dimensional time-lapse raw digital images (A) and experiment relevant inputs (B) are standardized into directories to facilitate throughput processing (C). Next, module 2 takes user-input parameters (D) to specify image processing algorithms (E) for image-based discrete oxidative stress measures over time (F). In module 3, PhagoSight algorithms are run from formatted directories for throughput cell tracking (G). User-input parameters (H) are then used to customize algorithms to control outcomes and visual tools for context (I). Lastly, in module 4 the information gathered from the previous three modules are consolidated into Excel databases that are automatically updated as more raw data is analyzed through the analysis pipeline (J and K).

Zebrafish inflammatory reactive oxygen species- and macrophage kinetics- based image analysis, Zirmi, software was developed to enable a pipeline for automated batch image processing and quantitative comparative analysis. The analysis software consisted of four modules: module 1, raw data formatting and standardization (**Fig. 2A-2C**); module 2, image-based fluorescence probe quantitation (**Fig. 2D-2F**); module 3, image-based cell fluorescence spatial and temporal migration kinetics (**Fig. 2G-2I**); module 4, data management and consolidation into Excel (**Fig. 2J** and **2K**). Additionally, supportive figures can be automatically exported to PowerPoint *via* Zirmi when applicable.

### MATLAB implementation

The Zirmi Software was developed in MATLAB R2015a. To streamline user inputs, graphical user interfaces were created to facilitate global access across modules. These user inputs were stored into databases within uniquely generated and amendable (.mat) files. Once stored, user inputs were used to standardize heterogeneous imaging as well as provide custom algorithm parameters (**Table S1**). The following imaging inputs were requested and used to standardize outcome measures: minutes post injury (MPI), sampling frequency (minutes per time frame), image lateral resolution (pixels per micron), z-stack resolution (microns per stack), and bits per pixel (BPP).

Fluorescent probe image analysis steps:

1. RED composite digital images were created by fusing all respective 2D sections together, scaling the intensities of each image jointly per time frame;
2. A median Gaussian filter was employed to remove Poisson noise present in confocal imaging due to a statistical variation in the number of detected photons;
3. Parameter 1 (pixel threshold) was defined to image segment fish tissue;
4. Morphological filters were employed using a kernel with a radius of 20 pixels, to erode spurious pixels;
5. Parameter 2 (ROI) was defined by outlining the wound margin in the BF image;
6. Parameter 3 (μm), the radial distance extended from the wound margin, was employed to define wound ROI area, inclusive of only fish tissue via image segmentation algorithms;
7. Parameter 4 (ROI), was defined as background region(s), selected, and averaged for a single background fluorescence intensity value “*B*” per respective time frame;
8. Background subtraction was employed, where “*I*” is the single wound ROI pixel intensity average value, per respective time frame, to acquire a single “*A*” value using the equation: *X* = *I*−*B*.
9. The single Integrated Density (IntDen) value, the wound ROI area “*A*” per respective time frame, was calculated with the equation: IntDen = *A* × *X*.

Fluorescent cell image analysis steps:

1. PhagoSight was employed: a detailed description of this method can be found at (http://phagosight.org) [20]. To avoid erroneous and manual tracking → Set GREEN image stack from 0 to 7 and BF as 8–9 → Set low and high threshold to optimize cell-to-cell segmentation and attenuate noise verified by visualization tools → Set minimum cell size surface area as 80 pixels^2^ → Save uniquely generated keyhole tracking file, “handles” into standard directories → Use visualization tools for quality control to verify distinguished tracks, and to eliminate collisions and artifacts.
2. Instantaneous speed, distance between tracks, net distance, max distance, and meandering index were algorithmically derived according to the literature [17];
3. tracks were further standardized with amendable parameters to minimize strenuous proofing at the user’s discretion→ parameter 5 (%), cell track inclusion criteria were based on the percent of distinguishable centroid position relative to time frame → parameter 6 (μm), maximum distance traveled by cell to be considered static → parameter 7 (centroid position, S_o_), epicenter of the wound gap or position of interest → parameter 8 (centroid position, N_o_,) notochord tip or position of interest, used to define S_1_ spatial domain → parameter 9 (μm), radial distance used to define spatial domains S_2_, S_3_ and S_4_;
4. direction based kinetics were derived from parameters → macrophage centroid positions were oriented with respect to S_o_, for discrete oriented net Euclid vector distances (μm) to wound determinations → Forward Migration Index (FMI), assigned 1 or 0, quantified the ratio discrete movements toward the “wound” → Forward to Backward Index (FBI), assigned 1 to −1, quantified as ((Forward movements—Backward movements)/Total movements).

### Statistical analysis

Statistical analysis of the data was performed by paired and unpaired Student *t*-test for parametric data *via* Microsoft Excel. Wilcoxon signed-rank test was used to test the hypothesis of zero medians for the difference between paired samples *via* MATLAB^®^. Differences were considered significant at *P* < 0.05.

## RESULTS

***Module 1: Data formatting of standardized three-dimensional time-lapse imaging***

The main goal of this software was to automate quantitative measures, for the analyses of thousands of images per fish, to determine quantitative discernable differences over time in oxidative stress (channel 1 fluorescence) and macrophage (channel 2 fluorescence) kinetics. A central limitation in throughput image analysis arises from inadvertent 3D+t imaging disturbances and unavoidable heterogeneity of batch-to-batch image acquisitions. To circumvent compromising data and time, assays were attuned to strict standardizations (**Fig. 1**). ROS probe loading and prolonged imaging preparation were performed in agreement with accepted and published experimental strategies [6,8]. Injury margins were created by “V”-shaped incisions within the caudal fin and were consistent in location and wound perimeter size. Digital greyscale images were formatted by channel (GREEN, RED, and BF) and archived into subdirectories according to batch, position of acquisition sequence (P0, P1, P2…, *etc*.), and time frame (T0001, T0002, T0003…, *etc*.). To permit greater throughput in analysis workflow (**Fig. 2**), image data was automatically organized into subdirectories by channel, z-stack, experiment batch, fish, and time frame. Lastly, (.mat) files were created with user-inputs (**Table S1**) to standardize future data measures regardless of heterogeneity of imaging.

***Module 2: Fluorescent probe image analysis***

**Figure 3.**
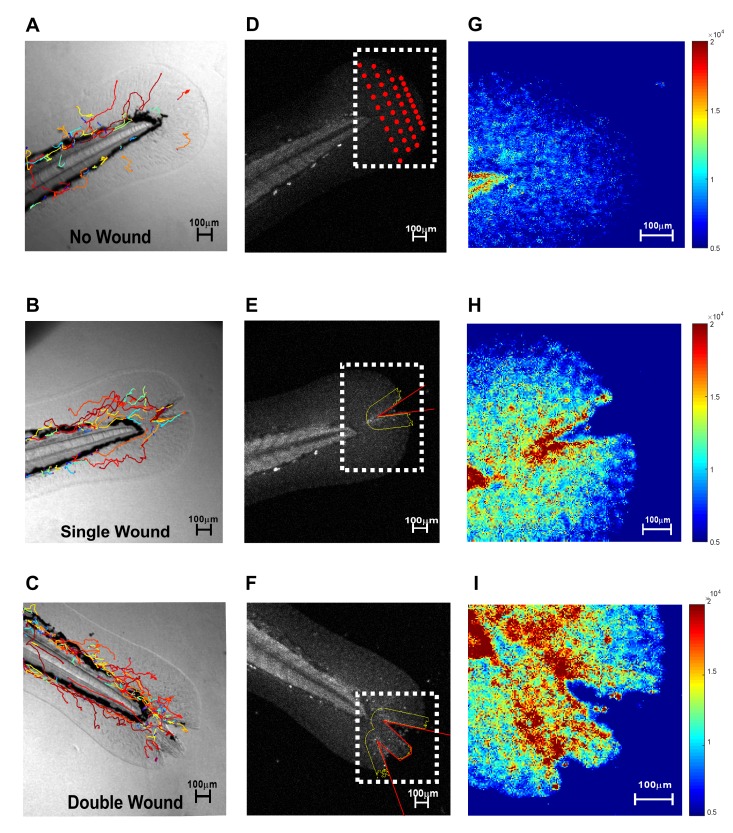
Zirmi module 2—reactive oxygen species probe image analysis. Macrophage migration patterns of unwounded control (A), single wounded (B), and double wounded (C) zebrafish tails can be detected, in parallel, with oxidative stress (D-F). In unwounded fins, 40 discrete 10 μm diameter circles are used to acquire background intensity fluorescence measures (D). In wounded fins, a uniform user-defined distance (yellow line, 65 μm) from wound perimeter (red line) is used to define wound areas (E and F). These defined wound regions of interest are used for throughput fluorescent intensity measures overtime, per fin injury type. These caudal fin conditions (dashed white boundary boxes) are also color-mapped (G-I) to provide qualitative insight on ROS probe intensity; red hue indicate high ROS signal and blue hue indicate low ROS signal.

The second module performed digital image analysis to compare fluorescent based measures between wound conditions (**Fig. 3A-3I**). Forty regions of interest pixel areas (ROIs, defined as 10-pixel diameter regions) were averaged to a single value for each unwounded fin per time frame. ROIs were determined *via* 40, 10-pixel diameter circles placed within unwounded caudal fin (**Fig. 3D**) that extended beyond the notochord. This enabled stochastic determinations of fluorescent intensity baseline values, to calculate the Integrated Density of the unwounded fin (see methods). To calculate a Corrected Total Fluorescence, CTF, measure for an unwounded fin, the Integrated Density and averaged background intensity value per respective time frame was incorporated in the following equation:

(1) *CTF of unwounded fin* = (*IntDen of unwounded fin*)**−(***A of unwounded fin* × *B*)

Next, for wounded fins, wound size and erroneous manual tracing biases needed to be eliminated. To circumvent manual tracing errors, V-shaped wounds were leveraged to accurately determine intensity values of interest. A 65 μm extended perimeter (yellow) surrounding the wound margin (red) (**Fig. 3E** and **3F**) was used to form a robust wound area value per respective time frame. To circumvent wound size bias, Integrated Density Correction was employed as previously established [21]. In this manner, the following equation was used to automate Corrected Total Fluorescence quantitative measures for a wounded fin:

(2) *CIF of unwounded fin* = (*IntDen of unwounded fin*)**−(***A of unwounded fin* × *X*

where “*X*” is the averaged background subtracted fluorescent intensity value per respective time frame. This standardized method for selecting wound region allowed for automated CTF measures on a time-lapse frame-by-frame basis. Zirmi pipeline further permitted ROI adjustments on a frame-by-frame basis whenever necessary to remain precise, mitigating small fin shifting. V-shape wounds with simple ROS probe strategies provided a uniform method for imaging analysis to standardize oxidative stress measures. **Figure 3** visualizations are automatically displayed in MATLAB and exportable, at the user’s discretion, to facilitate contextual understandings and to correct ROI’s in a meaningful way.

***Module 3: Fluorescent cell kinetic image-based spatial and temporal quantitation***

**Figure 4.**
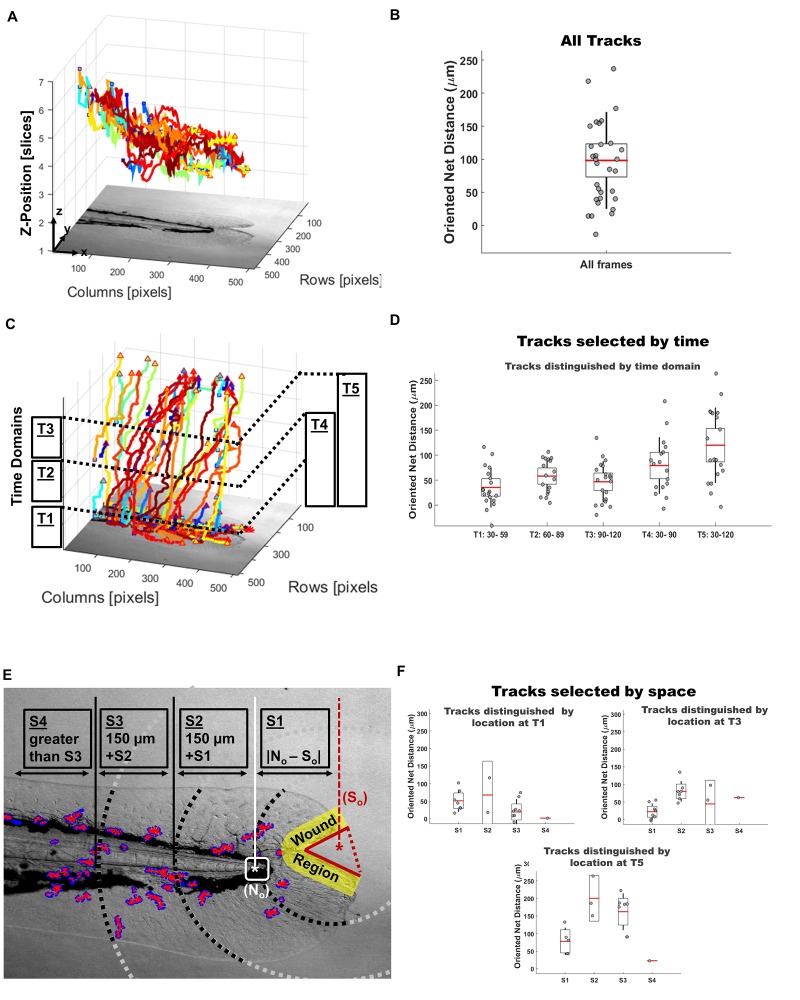
Zirmi module 3—cell migration image analysis. Macrophage discrete positions in the tail were tracked *via* PhagoSight keyhole modeling in a three-dimensional and time-lapse manner (A) to derive all cell tracks inclusive of all frames (B). With discrete position information, tracks were then parsed by time domains (C) to derive time-based changes (D). Selection of tracks by space was done by outlining the wound perimeter (red solid line) to get the wound epicenter (red asterisk) by which the user can use to segment by wound region (yellow) and additional space domains S1 (*via* white asterisk), S2, S3, and S4 (E). With these selection criteria, tracks could be grouped by time and space (F) on outcome measures ranging from speed, meandering index, static ratio, net distances, and direction. Selected tracks were plotted *via* MATLAB (B, D, F), exportable to PowerPoint.

The third module was designed to facilitate more uniform throughput in cell tracking and more granular cellular kinetic measures. Obtaining tracks is a labor-intensive process and requires several rounds of visual proofs to extract information worthy of comparative analysis. To tackle this obstacle a robust keyhole modeling solution, *via* PhagoSight, was implemented to determine macrophage centroid (3D+t) positions (**Fig. 4A** and **4B**) regardless of image-set size. To overcome constraints of single cumulative measures in one spatial or temporal domain [17], Zirmi adapted customizable algorithm parameters (**Table S1**). User-defined parameters permitted streamline proofing and quality control selection of tracks based on time and space. As a result, quantitative data measures incorporated descriptive metric outcomes, such as average speed, meandering index, and net distance toward the wound, to provide a simple and well-accepted mode of evaluating persistence and direction [17]. Alternative inferential measures require assumptions such as isotropic or ergodic conditions, relying heavily on finding the right representation of data; this can bottleneck reproducibility and minimize the utility of image data [22]. Zirmi adaptation of simple measures permitted automated elimination of confounders. For instance, if a cell centroid position was not identifiable at a time frame, then measures were equally absent from discrete and averaged data sets. This approach provided another dimension to proofing and selecting tracks, minimizing assumptions and saving time. To standardize track selection, a criterion was implemented to group tracks and respective measures according to user’s convenience. First, tracks that met a user-defined percent of distinguishable centroid positions were selected. This step mitigated the impact of measures from unequal track temporal duration; temporally shorter tracks can skew quantitative evaluations. Second, tracks were grouped according to time domains [T_1_ = 30–59 MPI; T_2_ = 60–89 MPI; T_3_ = 90–120 MPI; T_4_ = 30–90 MPI; T_5_ = 30–120 MPI] (**Fig. 4C** and **4D**). Last, tracks were grouped according to spatial domains [S_1_, S_2_, S_3_, S_4_,] originating from S_o_ (**Fig. 4E** and **4F**), as defined in Methods. Using Zirmi, customizable parameters allowed permutations of spatial and temporal domains for a more granular or cumulative comparative analysis. Measures that were grouped according to these track selection criteria, on a single fish basis, provided significant changes when compared to all-inclusive averages (**Table S2**).

### Validation and speed

A spatial overlap index, Dice Similarity Coefficient (DICE), was used to measure the reproducibility between manual and automated methods in regard to cell segmentations, fish tissue, and wound region over a 90-frame single wound imaging-set. DICE was adopted to validate various image segmentation. A value > 0.7 was considered to be effective overlap [23].

(3)
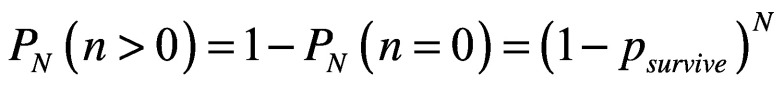


DICE reproducibility validation metric ranged from 0, no spatial overlap, to 1, complete overlap. We observed Zirmi automated image processing workflow to be as effective as manual image processing strategies with up to 10-fold increase in speed execution (**Table 1**). Manual methods were created in MATLAB scripts using single imaging processing workflows (**Script S1**). We observed manual image processing strategies of a reproducible “V”-shaped wound, as performed in Zirmi (**Script S2**), was difficult and highly prone to user error. Therefore, the execution speeds of the manual method for wound region was an interpretation of the time it would take to perform manual wound tracings at user’s best judgment (**Script S3**) compared to the Zirmi automated strategy. The DICE measure of the wound region represented the Zirmi image segmented spatial overlap accuracy between time frames and demonstrated effective overlap.

***Module 4: A representative experiment for database comparative analysis***

To test the assembly and utility of the Zirmi modular analysis pipeline, we compared differences in macrophage migration and oxidative stress, in parallel, based on wound severity, by which no current analysis pipeline exists (**Table 2**). Test data were gathered from three caudal fin wound severity conditions: unwounded (*n* = 4); single wound with a wound perimeter averaging a total length of 320 ± 10 μm, (*n* = 4); and doubly wounded with a wound perimeter, 544 ± 59 μm (*n* = 4, *P* < 0.0001), 1.7-fold larger than single wound perimeters. Imaging was performed with 90-frames (60 seconds sampling frequency) and three different channels (RED, GREEN, and BF) at 7 optical sections per frame (10 μm apart), resulting in 1890 images per fish analyzed in an automated manner. To facilitate standard uniform measures, wound regions were selected with a 65 μm radial distance from the wound perimeter (**Fig. 3D-3F**). Track selection consisted of 30 min based time domains (**Fig. 4C** and **4D**), with cell centroid positions detectable within 70% of frames. Tracks were oriented toward the epicenter of the wound gap (selected as S_o_, **Fig. 4E**). In unwound fish (baseline), the most distal position of the caudal fin was selected as S_o_. Briefly, we observed severity of wounding to be associated with quantitatively higher levels of ROS and more rapidly responding macrophages as demonstrated by increased velocities, decreased static activity, and increased recruitment to the wound sites over time (**Table 3**, **Fig. S1**). A 2- to 3-fold increase in ROS was observed when comparing unwounded (0.9 ± 0.2 × 10^7^) to singly wounded (2.2 ± 0.5 × 10^7^) caudal fin analyses. An additional 2- to 3-fold increase in ROS was observed from singly wounded to doubly wounded (5.4 ± 0.4 × 10^7^) caudal fins over time. In doubly wounded caudal fins, macrophage absolute velocities were significantly higher when compared to singly wounded (*P* = 0.02) at the initial time domain, or when compared unwounded fins at all time domains (*P* < 0.01). Static movements, defined as 0.9 μm or less, were measurably more prominent in uninjured caudal fins when compared to wounded fins in general. Overall, significant differences observed between interval and cumulative analysis emphasized the dynamic changes that can be easily detected in macrophage kinetics by track selection criteria and Corrected Total Fluorescence values.

## DISCUSSION

The decision to create an open-source image analysis pipeline, Zirmi, was based on a lack of readily available and customizable software solutions capable of automating a quantitative assessment of fluorescence outcome measures in the zebrafish model. Zirmi prompted user-accessible interfaces to customize algorithm parameters by which users could specify or balance the quality of measures relevant to their investigative purposes. To demonstrate the utility of the Zirmi image analysis pipeline, our experimental results, based on wounding severity and oxidative stress, provided additional detail to previous studies defining early wound macrophage recruitment [7,15,16,19]. These findings emphasize the importance of standardized automated analysis pipelines for experimental conditions, such as wound size, for accurate evaluations of oxidative stress and macrophage migration.

To develop an automated process, we defined attributes of the wound for a standardized approach. We used a “V”-shaped incision within the caudal fin as a necessary approach towards standardization of the wound. This approach differed from other caudal fin wounding methods, such as amputations applied perpendicularly to the notochord line. “V”-shape wounds provided a reproducible and measurable injury that imaging processing tools could use to standardize both wound oxidative stress and cell kinetic-based outcome measures. In prior experiments, we observed caudal fin amputations to result in a large single wound line, which made tracking and localizing cells within the wound proximity problematic. Wound orientation was too broad and cell clustering was increased making it difficult to algorithmically separate and track cells (data not shown). Another standardization was the definition of the wound perimeter. We observed that a larger wound margin consistently demonstrated a larger inflammatory response via increased ROS signal and increased macrophage activity. To allow automated comparisons between experiments, wound perimeters of the same treatment group were defined within a prescribed range; we believe that these boundaries set automatically improved the accuracy and reproducibility of our measures. Other standardizations included the use of non-wounded fish to serve as standardized, baseline ROS calculations.

DHE has been reported as a reliable quantitative measure of ROS in other applications [6,8]. Our automated approach enabled quantitative real time measurements, which is an advantage over existing methods that have been accomplished *ex vivo*, either through proteomic assays [6], flow cytometry [8] or through HyPer genetically encoded peroxide levels [5]. Jelcic *et al*. have reported an image-based software approach, incorporating multiple software packages to elegantly quantify hydrogen peroxide reaction-diffusion kinetics in zebrafish [24]. This method provides a unique and sensitive means to measure H_2_O_2_ diffusion in real time using a radiometric biosensor. Our simplicity of method, which also quantifies ROS in real time, differs in that its method is designed to work with higher throughputs and is formatted to work with cell tracking. We observed Corrected Total Fluorescent values to be comparable across fish showing consistent, uniform, and reproducible results. Thus, CTF allowed for comparative analysis of fish with differing wound severity. To address confounding areas of expression, we applied user proofing and manual tracing. To address the potential confounding detection of DHE within the notochord, uniform wound distances of 65 μm were defined as the limits of wound boundaries. In future applications, as fluorescent probes continue to improve in specificity [25], we expect simple Zirmi open-source image-based quantitative methods to become even more effective.

Established PhagoSight, pre-processing and keyhole algorithms, were integrated into Zirmi workflow to leverage current powerful existing solutions and to avoid parallel developments. PhagoSight keyhole modeling algorithms can provide data on the most probable landing position of a macrophage in a time-ordered set of coordinate points [18]. This software has advanced the field in providing single measures in one space or time domain [17]. Such analytics are time-consuming and may require several rounds of visual proofs for comparative analysis. To increase throughput, we have extended this knowledge base by adding standardized methods. Zirmi software eliminates uncertainties from computations if not proofed and corrected by the user. This approach contrasts to the more inferential measures of migration persistence and direction bias that have been used, such as, mean squared displacement, turning angle distribution, tangent to tangent correlation, and persistent random walks (PRW) [26]. Because these measures make inferential determinations implicitly dependent on the sampling interval, they are not reproducible without equivalent sampling intervals across every analysis and high fidelity of cell positions [22]. The advantage of Zirmi is that it incorporates a standardized workflow to eliminate confounding effects of heterogeneous batch imaging conditions, such as varying sampling acquisition.

Custom made non-generic image solutions with greater user control and reliability are needed to answer specific biological questions in the zebrafish whole organism model [9]. The contribution of this work to the field is its ability to automatically process three-dimensional time-lapse images of both oxidative stress and macrophage migration over time for comparative analysis. Defining early events of macrophage activity and their degree of response to ROS can provide critical insights for the development of wound therapeutics. Future studies will incorporate this method to define simultaneous changes in ROS and macrophage metrics and define to what extent these parameters are perturbed in normal and dysfunctional wound healing conditions.

## Supplementary Material

Supplementary information Zirmi open-source can be accessed at the following URL: https://sites.google.com/uic.edu/bartholomewlab/resources. A repository was created on Github URL: https://github.com/ADParedes/Zirmi, where example data and open-source scripts from this paper are available. A PhagoSight repository can be found at Github URL: https://github.com/phagosight/phagosight).
**Figure S1.** Representative plots of experimental groups in **Table 3**.
**Table S1.** User-defined input parameters incorporated into the Zirmi analysis pipeline.
**Table S2.** Macrophage time-based changes of velocity and static ratio in a single wound case-study.
**Script S1.** A MATLAB script of a single image segmentation workflow.
**Script S2.** A MATLAB script of a single image automated “V” shaped segmentation workflow.
**Script S3.** A MATLAB script of a single image manual tracing segmentation workflow.
Supplementary information of this article can be found online at
http://www.jbmethods.org/jbm/rt/suppFiles/259.

## Figures and Tables

**Table 1.** Accuracy verification and execution time of Zirmi image analysis pipeline.

	Accuracy verification	Zirmi execution speed	
Time-lapse image segmentation	DICE	Time automatic (s)	Time manual (s)
Module 2 wound region	0.84. ± 0.07	160 ± 16	2 020 ± 157
Module 2 caudal fin tissue	0.90 ± 0.18	122 ± 10	5 110 ± 233
Module 3 macrophages	0.78 ± 0.2	977 ± 512	3 740 ± 912

**Table 2.** Advances in real time quantitative simultaneous cell tracking and ROS imaging.

First author	Cell tracking method	Quantifiable cell tracking	Automated cell tracking	ROS imaging	Quantifiable *in vivo* ROS measure	Automated ROS measure
Koopman *et al*. [10]	Not available	Not available	Not available	Single cell *ex vivo*, flow cytometric; real time unavailable	Not available	Not available (single timepoint measures)
Owusu-Anshah *et al*. [8]	Not available	Not available	Not available	Dihydroethidium fluorescent probe for real time live tissue imaging in Drosophila	Not available	Not available (single qualitative measures)
Hall *et al*. [7]	Image J	No solution provided (requires additional software)	Time-lapse cell tracking; additional tools required for visual proofing	Not available	Not available	Not available
Pase *et al*. [12]	Not available	Not available	Not available	Metamorph fluorescent HyPer H_2_O2 imaging	HyPer fluorescent intensity ratio measure	Not available
Walker *et al*. [11]	Not available	Not available	Not available	No image-based solution available; relies on fluorescent microplate reading; cannot detect wound region	ARQiv microplate whole fish detection	High throughput
Henry *et al*. [20]	PhagoSight, MATLAB	Quantitative with data checking feature	Open-source, package; automated method for cell tracking and single time domain data measures	Not available	Not available	Not available
Mugoni *et al*. [6]	Not available	Not available	Not available	Single cell *ex vivo* fluorescent probe based	Not available (flow cytometric based)	Not available; (single timepoint measures)
Wang *et al*. [2]	Single image- based cell counts	Not available	Not available	Not available; PCR based	Not available	Not available
Zhang *et al*. [25]	Not available	Not available	Not available	Single cell and whole fish fluorescent-probe based imaging	Fluorescent-intensity – method not provided	Not available; (single time point measures)
Jelcic *et al*. [24]	Not available	Not available	Not available	Real time image-based measures of HyPer within zebrafish wounds	Corrected HyPer fluorescent intensity ratio-based measures	Image J, MATLAB, and Python methods used for specific automation

**Table 3.** Comparison of group zebrafish average ROS generation & average Macrophage velocity, static ratio, and wound recruitment.

Time frame (min)	Zirmi 30–59	Zirmi 60–89	Zirmi 90–120	Zirmi 45	Zirmi 60	Zirmi 90	Zirmi 120
	Velocity [μm/min]	Velocity [μm/min]	Velocity [μm/min]	Mφ # at wound	Mφ # at wound	Mφ # at wound	Mφ # at wound
NW	3.07 ± 0.06	2.88 ± 0.40	2.70 ± 0.40	NA	NA	NA	NA
SW	3.65 ± 0.4	4.04 ± 0.14	4.27 ± 0.48	2 ± 1	4 ±1	5 ± 1	6 ± 1
DW	4.43 ± 0.35	4.21 ± 0.20	4.12 ± 0.21	4 ± 1	6 ± 2	7 ± 1	8 ± 1
	*P* values						
NW *vs*. SW	*P = 0.049	**P = 0.003	**P = 0.006	NA	NA	NA	NA
NW *vs*. DW	**P = 0.003	*P = 0.005	**P = 0.001	NA	NA	NA	NA
SW *vs*. DW	*P = 0.02	NS *P* = 0.2	NS *P* = 0.3	*P = 0.03	NS *P* = 0.06	*P = 0.04	*P = 0.04
	Static ratio (0 to 1)	Static ratio (0 to 1)	Static ratio (0 to 1)	CTF 10^6^	CTF 10^6^	CTF 10^6^	CTF 10^6^
NW	0.60 ± 0.21	0.59 ± 0.20	0.61 ± 0.19	0.91 ± 0.27	0.90 ± 0.21	0.9 ± 0.24	0.94 ± 0.2
SW	0.32 ± 0.1	0.23 ± 0.08	0.25 ± 0.04	2.34 ± 0.72	2.17 ± 0.53	2.13 ± 0.4	2.15 ± 0.2
DW	0.18 ± 0.07	0.20 ± 0.07	0.24 ± 0.07	5.92 ± 0.32	5.58 ± 0.4	5.22 ± 0.5	5.0 ± 0.28
	*P* values						
NW *vs*. SW	*P = 0.01	*P = 0.01	*P = 0.01	*P = 0.03	*P = 0.02	**P = 0.004	**P = 0.001
NW *vs*. DW	*P = 0.01	*P = 0.01	*P = 0.02	***P < 0.0001	***P < 0.0001	***P <0.0001	NA
SW *vs*. DW	*P = 0.04	NS *P* = 0.3	NS *P* = 0.4	*P = 0.04	**P = 0.001	***P = 0.0001	***P < 0.0001

NW, no wound; SW, single wound; DW, double wound; *M*φ, *macrophage*; #, number; NA, not applicable; NS, not significant. Unpaired *t*-test (

*P < 0.05,

**P < 0.01,

***P < 0.001, NS > 0.05).

## References

[1] Ross R Odland G (1968) Human wound repair. II. Inflammatory cells, epithelial-mesenchymal interrelations, and fibrogenesis. J Cell Biol 39: 152-168. doi: 10.1083/jcb.39.1.152. PMID: 5678446 PMC2107500

[2] Wang Q Liu S Hu D Wang Z Wang L (2016) Identification of apoptosis and macrophage migration events in paraquat-induced oxidative stress using a zebrafish model. Life Sci 157: 116-124. doi: 10.1016/j.lfs.2016.06.009. PMID: 27288846

[3] Baker M (2011) Screening: the age of fishes. Nat Methods 8: 47-51. doi: 10.1038/nmeth0111-47. PMID: 21191372

[4] Forn-Cuní G Varela M Pereiro P Novoa B Figueras A (2017) Conserved gene regulation during acute inflammation between zebrafish and mammals. Sci Rep 7: 41905-1038. doi: 10.1038/srep41905. PMID: 28157230 PMC5291205

[5] Niethammer P Grabher C Look AT Mitchison TJ (2009) A tissue-scale gradient of hydrogen peroxide mediates rapid wound detection in zebrafish. Nature 459: 996-999. doi: 10.1038/nature08119. PMID: 19494811 PMC2803098

[6] Mugoni V Camporeale A Santoro MM (2014) Analysis of oxidative stress in zebrafish embryos. J Vis Exp 2014: 10-3791. doi: 10.3791/51328. PMID: 25046434 PMC4212721

[7] Hall C Flores MV Crosier K Crosier P (2009) Live cell imaging of zebrafish leukocytes. Methods Mol Biol 546: 255-271. doi: 10.1007/978-1-60327-977-2_16. PMID: 19378109

[8] Owusu-Ansah E Yavari A Banerjee U (2008) A protocol for in vivo detection of reactive oxygen species.Protocol Exchange. doi: 10.1038/nprot.2008.23.

[9] Mikut R Dickmeis T Driever W Geurts P Hamprecht FA (2013) Automated processing of zebrafish imaging data: a survey. Zebrafish 10: 401-421. doi: 10.1089/zeb.2013.0886. PMID: 23758125 PMC3760023

[10] Koopman WJH Verkaart S van Emst-de Vries E. (Sjenet) Grefte S Smeitink JAM (2006) Simultaneous quantification of oxidative stress and cell spreading using 5-(and-6)-chloromethyl-2′,7′-dichlorofluorescein. Cytometry A 69: 1184-1192. doi: 10.1002/cyto.a.20348. PMID: 17066472

[11] Walker SL Ariga J Mathias JR Coothankandaswamy V Xie X (2012) Automated reporter quantification in vivo: high-throughput screening method for reporter-based assays in zebrafish. PLoS One 7: 10-1371. doi: 10.1371/journal.pone.0029916. PMID: 22238673 PMC3251595

[12] Pase L Nowell CJ Lieschke GJ (2012) In vivo real-time visualization of leukocytes and intracellular hydrogen peroxide levels during a zebrafish acute inflammation assay. Methods Enzymol 506: 135-156. doi: 10.1016/B978-0-12-391856-7.00032-9. PMID: 22341223

[13] Schindelin J Arganda-Carreras I Frise E Kaynig V Longair M (2012) Fiji: an open-source platform for biological-image analysis. Nat Methods 9: 676-682. doi: 10.1038/nmeth.2019. PMID: 22743772 PMC3855844

[14] Schneider CA Rasband WS Eliceiri KW (2012) NIH Image to ImageJ: 25 years of image analysis. Nat Methods 9: 671-675. doi: 10.1038/nmeth.2089. PMID: 22930834 PMC5554542

[15] Li L Yan B Shi Y Zhang W Wen Z (2012) Live imaging reveals differing roles of macrophages and neutrophils during zebrafish tail fin regeneration. J Biol Chem 287: 25353-25360. doi: 10.1074/jbc.M112.349126. PMID: 22573321 PMC3408142

[16] Gray C Loynes CA Whyte MKB Crossman DC Renshaw SA (2011) Simultaneous intravital imaging of macrophage and neutrophil behaviour during inflammation using a novel transgenic zebrafish. Thromb Haemost 105: 811-819. doi: 10.1160/TH10-08-0525. PMID: 21225092

[17] Svensson C Medyukhina A Belyaev I Al-Zaben N Figge MT (2017) Untangling cell tracks: Quantifying cell migration by time lapse image data analysis. Cytometry A 93: 357-370. doi: 10.1002/cyto.a.23249. PMID: 28976646

[18] Reyes-Aldasoro CC Akerman S Tozer GM (2008) Measuring the velocity of fluorescently labelled red blood cells with a keyhole tracking algorithm. J Microsc 229: 162-173. doi: 10.1111/j.1365-2818.2007.01877.x. PMID: 18173654

[19] Ellett F Pase L Hayman JW Andrianopoulos A Lieschke GJ (2010) mpeg1 promoter transgenes direct macrophage-lineage expression in zebrafish. Blood 117: doi: 10.1182/blood-2010-10-314120. PMID: 21084707 PMC3056479

[20] Henry KM Pase L Ramos-Lopez CF Lieschke GJ Renshaw SA (2013) PhagoSight: an open-source MATLAB® package for the analysis of fluorescent neutrophil and macrophage migration in a zebrafish model. PLoS One 8: 2013-8. doi: 10.1371/journal.pone.0072636. PMID: 24023630 PMC3758287

[21] McCloy RA Rogers S Caldon CE Lorca T Castro A (2014) Partial inhibition of Cdk1 in G 2 phase overrides the SAC and decouples mitotic events. Cell Cycle 13: 1400-1412. doi: 10.4161/cc.28401. PMID: 24626186 PMC4050138

[22] Loosley AJ O'Brien XM Reichner JS Tang JX (2015) Describing directional cell migration with a characteristic directionality time. PLoS One 10: 10-1371. doi: 10.1371/journal.pone.0127425. PMID: 25992908 PMC4439174

[23] Zou KH Warfield SK Bharatha A Tempany CMC Kaus MR (2004) Statistical validation of image segmentation quality based on a spatial overlap index1 scientific reports. Acad Radiol 11: 178-189. doi: 10.1016/s1076-6332(03)00671-8. 14974593 PMC1415224

[24] Jelcic M Enyedi B Xavier JB Niethammer P (2017) Image-Based Measurement of H2O2 Reaction-Diffusion in Wounded Zebrafish Larvae. Biophys J 112: 2011-2018. doi: 10.1016/j.bpj.2017.03.021. PMID: 28494970 PMC5425381

[25] Zhang R Zhao J Han G Liu Z Liu C (2016) Real-time discrimination and versatile profiling of spontaneous reactive oxygen species in living organisms with a single fluorescent probe. J Am Chem Soc 138: 3769-3778. doi: 10.1021/jacs.5b12848. PMID: 26938117

[26] Liepe J Sim A Weavers H Ward L Martin P (2016) Accurate Reconstruction of Cell and Particle Tracks from 3D Live Imaging Data. Cell Syst 3: 102-107. doi: 10.1016/j.cels.2016.06.002. PMID: 27453447 PMC4963212

